# Editorial: Pre-cueing Effects on Perception and Cognitive Penetrability

**DOI:** 10.3389/fpsyg.2018.00230

**Published:** 2018-02-27

**Authors:** Athanassios Raftopoulos, Gary Lupyan

**Affiliations:** ^1^Department of Psychology, University of Cyprus, Nicosia, Cyprus; ^2^Lupyan Lab, Psychology, University of Wisconsin-Madison, Madison, WI, United States

**Keywords:** pre-cueing, attention, cognitive penetrability, mental imagery, early vision

Attention has often been likened to spotlights and filters that illuminate or screen out some inputs in favor of others. This, largely passive, conception of attention has been gradually replaced by a dynamic and far-reaching process. Attention augments neural processing at all levels. Attention contributes to testing hypotheses concerning the distal causes of the sensory data encoded in the lower neuronal assemblies. This testing assumes the form of matching predictions made on the basis of an hypothesis, about the sensory information that the lower levels should encode if the hypothesis is correct, with the actual sensory information encoded at the lower levels. To this aim, attention enhances or sharpens the activity of neurons in the cortical regions that encode the stimuli that most likely contain information relevant to this testing.

Concerning pre-cueing, studies of spatial and feature/object attention cues show early modulation of pre-stimulus activity in the visual cortex. Attention cueing can function in flexible and complex ways: people can be cued to attend to various objects, properties, and semantic categories and such attention appears to involve directly early perceptual mechanisms. This phenomenon refers to the enhancement of the baseline activity of neurons in the visual cortex that are tuned to the cued location or code the cued feature(s).

In this Research Topic, we aim to answer two questions: First, how do attentional cuing effects relate to top–down influences on perception? Second, given that in pre-cueing cognitively driven attention appears to change perceptual processing, does the pre-cueing attentional modulation ental the cognitive penetrability of perception? Addressing these two questions will shed light on the theoretical underpinnings of cognitive penetrability and the role of attention.

Feng and Spence examine how endogenous spatial pre-cues influence the allocation of attention in the periphery of the visual field. They present two experiments that examine how the expectation of the target's location shapes the distribution of attention across various eccentricities. Their findings suggest that spatial pre-cueing results in higher target detection rates and that a higher target detection rate is found when the target occurred at the cued direction. These findings evidence the cognitive penetrability of early vision.

Lammers et al. distinguish two conceptions of cognitive penetrability. In the broad sense, attention and memory are not pre- and post-perceptual systems but parts of the mechanisms by which top-down processes influence perception. In the narrow sense, cognitive penetrability only occurs when top–down factors are flexible and cause an illusion. Since one cannot be cognitively trained to see and unsee illusions, illusions cannot be driven by cognition in the narrow sense. However, most research focuses on foveal vision that is too unambiguous for cognitive factors to control perception. Illusions in more ambiguous peripheral visual perception could offer a different insight into this problem.

Wu and Zhao focus on prior knowledge of object associations as an aspect of attentional selection and review recent studies demonstrating that how objects are selected depends on the participant's prior experience with other objects associated with the target. Thus, prior knowledge of the test and related stimuli acquired before or during the task impacts performance since it affects attentional selection and information acquisition. Wu and Zhao do not discuss whether the effects of prior knowledge of object associations on perception constitute cases of cognitive penetrability.

Montemayor and Haladjian argue that the opposing views that cognitive penetration is pervasive and that there is a fundamental distinction between cognition and perception, which precludes cognitive penetration, are too extreme, but both theories have merits and empirical support. To address this puzzle, they discuss a theoretical approach that incorporates the merits of these two views into a broader and more nuanced explanatory framework, the consciousness and attention dissociation framework that they have developed in previous work.

Lupyan addresses two arguments aim to exclude attention from signifying the cognitive penetrability of perception. That attention is a post-perceptual process reflecting selection between fully constructed perceptual representations, and that attention is a pre-perceptual process that selects the input to encapsulated perceptual systems. Lupyan argues that although some attentional effects can be construed as post-perceptual, and that spatial attention can be seen as selecting the input, other forms of attention operate so as to change perceptual content across the entire visual hierarchy; attention is one of the mechanisms by which cognition affects perception.

Fazekas and Nanay focus on pre-cueing effects in early vision. They argue that the claim that pre-cueing studies show that perception is cognitively penetrated by means of attentional mechanisms is problematic. They argue, however, that pre-cueing studies show that perception is cognitively penetrated via mental imagery. Cue-induced mental imagery provides a channel through which cognitive states can exert such effects on perception that fulfill the requirements of cognitive penetration.

Gross notes that Pylyshyn argues that cognitively driven attentional effects do not amount to cognitive penetration of early vision because such effects occur either before or after early vision. Critics object that such effects occur at all levels of perceptual processing but Gross supports Pylyshyn's claim. Even if Pylyshyn's critics are correct that attentional effects are not external to early vision, these effects do not satisfy Pylyshyn's requirements that the effects be direct and exhibit semantic coherence for cognitive penetration to occur.

Gatzia and Brogaard argue that it is usually assumed that covert endogenous attention differs significantly from overt endogenous attention. However, studies indicate that the oculomotor system is activated when covert attention is directed to an uncued location suggesting that covert endogenous attention may involve attentional shifts, albeit less apparent than the shifts in overt attention. The differences in the perceptual outputs could, thus, be attributed to selectively attending to a different object or a different feature of the same object. The effects of covert attention, then, can be attributed either to processes that resemble perceptual learning or attentional shifts that are not cases of cognitive penetration.

Finally, Raftopoulos defends the cognitive impenetrability of early vision in view of pre-cueing effects. He discusses the problems that cognitive penetrability causes for the epistemic role of perception in grounding perceptual beliefs and he argues that perceptual processes are cognitively penetrable if the cognitive effects undermine their epistemic role. He argues then that the cognitive effects that act through pre-cueing do not undermine the epistemic role of early vision and, also, they do not affect early vision directly; early vision is cognitively impenetrable.

The chapters in this volume show why the effects of attention on perceptual processing in general and the nature of pre-cueing in particular have attracted so much attention in the last two decades. The ever-increasing empirical literature is very rich and amenable to a variety of interpretations and, thus, its implications are hotly debated both in Philosophy and the Cognitive Sciences.

## Author contributions

All authors listed have made a substantial, direct and intellectual contribution to the work, and approved it for publication.

### Conflict of interest statement

The authors declare that the research was conducted in the absence of any commercial or financial relationships that could be construed as a potential conflict of interest.

